# NO-sensitive guanylyl cyclase is expressed in pericytes but absent from endothelial cells in the murine lung

**DOI:** 10.1186/1471-2210-11-S1-P38

**Published:** 2011-08-01

**Authors:** Peter König, Dieter Groneberg, Roland Jäger, Andreas Friebe

**Affiliations:** 1 Institut für Anatomie, Universität zu Lübeck, Lübeck, Germany; 2 Physiologisches Institut, Julius-Maximilians-Universität, Würzburg, Germany

## Background

In the lung, nitric oxide (NO) regulates vascular tone and permeability via its receptor NO-sensitive guanylyl cyclase (NO-GC). Although data from vascular beds of other species imply that NO-GC is expressed in microvascular endothelial cells, definitive information of its cellular expression in the alveolar region is lacking.

This study aimed to clarify the expression of NO-GC in the lung by using an antibody specific for the β1 subunit of NO-GC as judged by absence of staining in NO-GC-deficient mice [1,2]. Immunohistochemistry was performed in murine lungs using 200 µm thick precision cut lung slices (PCLS) and frozen sections for preembedding staining on the EM level. In addition to NO-GC, the expression of phosphodiesterase 5 (PDE5) was determined by immunohistochemistry. Furthermore, cGMP production was visualized using a cGMP-specific antibody after NO stimulation of PCLS in the absence and presence of the PDE5 inhibitor sildenafil. Activity and expression of NO-GC in cultured microvascular murine lung endothelial cells were determined by radioimmunoassay of NO-induced cGMP synthesis and Western blotting, respectively.

## Results

NO-GC immunoreactivity was detected in vascular smooth muscle cells as anticipated from functional studies. In the alveolar region, however, NO-GC staining was restricted to cells in the alveolar wall; these cells were distinct from endothelial cells and exhibited highly branched processes. These processes were in close contact with endothelial cells. Close to the pleural surface where alveolar capillaries tend to be more elongated, NO-GC-immunoreactive cells had the classical morphology of pericytes. Electron microscopy revealed that NO-GC-immunoreactive processes of highly branched cells shared the basement membrane with endothelial cells which is characteristic for pericytes. Cells with the same morphology as NO-GC-immunoreactive cells exhibited PDE5 immunoreactivity. Indeed, in the presence of sildenafil, NO stimulation of PCLS resulted in cGMP-specific immunostaining in the highly branched NO-GC-immunoreactive cells. Stimulation of PCLS in the absence of PDE inhibitor did not result in any detectable cGMP. Endothelial cells did not reveal any immunoreactivity for NO-GC, cGMP or PDE5. In addition, NO-GC was not detected in cultured murine lung microvascular endothelial cells by Western blotting and, in line with this, NO-induced cGMP synthesis could not be measured in these cells.

## Conclusion

Our results indicate that NO-GC is expressed in pericytes but not in endothelial cells in the mouse lung. It is therefore conceivable that NO-driven changes in lung microvascular permeability are mediated by pericytes.
